# Triple Rule Out versus CT Angiogram Plus Stress Test for Evaluation of Chest Pain in the Emergency Department

**DOI:** 10.5811/westjem.2015.6.25958

**Published:** 2015-10-20

**Authors:** Kelly N. Sawyer, Payal Shah, Lihua Qu, Michael C. Kurz, Carol L. Clark, Robert A. Swor

**Affiliations:** * William Beaumont Hospital, Department of Emergency Medicine, Royal Oak, Michigan; † William Beaumont Hospital, Research Institute Center for Outcomes Research, Royal Oak, Michigan; ‡ University of Alabama School of Medicine, Department of Emergency Medicine, Birmingham, Alabama

## Abstract

**Introduction:**

Undifferentiated chest pain in the emergency department (ED) is a diagnostic challenge. One approach includes a dedicated chest computed tomography (CT) for pulmonary embolism or dissection followed by a cardiac stress test (TRAD). An alternative strategy is a coronary CT angiogram with concurrent chest CT (Triple Rule Out, TRO). The objective of this study was to describe the ED patient course and short-term safety for these evaluation methods.

**Methods:**

This was a retrospective observational study of adult patients presenting to a large, community ED for acute chest pain who had non-diagnostic electrocardiograms (ECGs) and normal biomarkers. We collected demographics, ED length of stay, hospital costs, and estimated radiation exposures. We evaluated 30-day return visits for major adverse cardiac events.

**Results:**

A total of 829 patients underwent TRAD, and 642 patients had TRO. Patients undergoing TRO tended to be younger (mean 52.3 vs 56.5 years) and were more likely to be male (42.4% vs. 30.4%). TRO patients tended to have a shorter ED length of stay (mean 14.45 vs. 21.86 hours), to incur less cost (median $449.83 vs. $1147.70), and to be exposed to less radiation (median 7.18 vs. 16.6mSv). No patient in either group had a related 30-day revisit.

**Conclusion:**

Use of TRO is feasible for assessment of chest pain in the ED. Both TRAD and TRO safely evaluated patients. Prospective studies investigating this diagnostic strategy are needed to further assess this approach to ED chest pain evaluation.

## INTRODUCTION

Chest pain is one of the most common reasons people seek medical attention in the emergency department (ED). Evaluation of non-specific chest pain often requires testing for different life-threatening clinical entities, such as aortic dissection, pulmonary embolism, or acute coronary syndrome (ACS). Several clinical and non-invasive stratification tools have been explored to avert missed cardiovascular emergency diagnoses.^1^–^11^ Still, with an aging population and a dramatic increase in the number of ED visits overall, the frequency of this vexing presentation will only increase.^12^–^16^

Chest pain and observation units have evolved for further risk stratification of low and intermediate risk patients.^17^–^19^ These units typically use supplemental objective testing to exclude ACS, as well as other life-threatening emergencies. Testing strategies include chest computed tomography (CT) with varying protocols for aortic dissection and pulmonary or coronary angiography, as well as stress testing, either alone or more typically using myocardial perfusion imaging (MPI) or stress echocardiography. While these observation units are safe and effective while reducing cost and length of stay, there is no direct evidence that this strategy reduces adverse cardiac events.^20^–^22^

CT coronary angiography has recently been implemented as a diagnostic tool in low-risk patients for coronary artery disease.^23^–^25^ A variant of this method, CT coronary angiography with triple rule out protocol (triple rule out, TRO), has been used to evaluate the presence of coronary artery disease, as well as pulmonary embolism and aortic dissection.^26^,^27^ This test, however, draws controversy, in part because of concerns regarding the technique used and the performance characteristics of this test.^28^–^30^ Critics argue that the pre-test probability for each of these three causes of chest pain is never equal enough to warrant TRO protocol.^31^ Yet, typically two etiologies (most commonly ACS and pulmonary embolism) are often considered plausible, and providers not infrequently apply a serial approach to evaluation, including first a chest CT pulmonary embolism protocol and then subsequent provocative testing (TRAD), often graded exercise stress with MPI.^32^ While TRO has been demonstrated as safe and effective as a diagnostic strategy for ACS, no literature exists to compare the safety and effectiveness of these two approaches.^33^

For these reasons, we reviewed the ED course and 30-day outcome for patients with undifferentiated chest pain evaluated with TRO compared to patients who received TRAD. We hypothesized that both methodologies would be safe and effective for ED patients.

## METHODS

### Study Setting & Population

Our study was performed in a single academic community hospital with 1,066 inpatient beds and 115,000 annual ED visits. It was approved by our institutional review board as an expedited review with a waiver of informed consent.

This was a retrospective observational cohort study whereby the testing strategy was at the discretion of the treating physician and thus no pre-test probability assessment is available. We collected demographics and process data from the electronic medical record, including age, gender, body mass indexes, total and ED length of stay (hours), and short-term revisit details. Data abstraction was conducted electronically by our experienced outcomes research director (LQ).

All adult patients (age≥18), evaluated initially in the ED for chest pain between February 2009 and January 2012, were considered for inclusion if they had one of two testing strategies: 1) Coronary CTA-TRO protocol or 2) dedicated Chest CT and provocative testing for ischemic cardiac disease (TRAD). We excluded patients who had abnormal biomarkers, abnormal ECGs, a single imaging study to evaluate for the cause of chest pain, an initial abnormal chest CT, or a high suspicion of ACS warranting admission to the hospital.

Testing strategy was almost exclusively chosen by the ED physician alone. Both TRO and stress testing were available seven days per week during business hours, with the exception of stress echocardiography, which was not available on weekends. Patients who presented late in the day required transfer to the ED observation unit until evaluation could be completed. In these cases, the treating ED physician still determined the testing strategy to be carried out in observation, with rare changes to individual plans based on patient factors (i.e. inability to beta block sufficiently) rather than consultant input. Ultimately ED providers made decisions regarding further testing, discharge, or admission from the observation unit.

#### Measurements & Outcomes

Total hospital costs (dollars) include direct and variable patient care costs, but did not include physician professional fees. We calculated total cost using Sunrise ESPI software. Radiation doses (mSv) were estimated by radiation physicists in both nuclear medicine and imaging, based on average radiation dosing for each study and patient body mass index.^34^ TRO protocol used a triphasic injection, with 100mL of contrast at 5mL/sec, then an additional 30mL at 3mL/sec to maintain pulmonary artery opacification, followed by a standard saline injection. TRO images were acquired in a caudal-cranial fashion.

Our measure of safety was revisit for major adverse cardiac event (MACE), including death, acute myocardial infarction, and revascularization, or venous thromboembolic disease within 30 days of the initial admission. All patients were reviewed for 30-day revisits to our health system, and revisit details were manually reviewed by two independent study authors, blinded to testing strategy, to identify whether revisits met adverse event criteria or were unrelated to the index visit.

#### Data Analysis

Data are presented as means and standard deviations if normally distributed, or medians and inter-quartile ranges if non-normal. No statistical inferences were made. We used the software JMP 9.0.2 (SAS Inc., Cary, NC) to calculate descriptive statistics.

## RESULTS

This study investigated two populations (Figure): 829 patients who were evaluated using TRAD and 642 patients who were evaluated using TRO.

Demographics by group are presented in Table 1 and radiation estimates based on body mass index are shown in Table 2. TRO patients were younger (mean 52.3 versus 56.5 years); had lower body mass index (mean 29.4 versus 31.8); and were more likely to be male (42.4% versus 30.4%). TRO patients also incurred less cost (median $449.83 versus $1147.70) and less radiation exposure (median 7.18mSv versus 16.6mSv). For the TRO cohort, eight patients were found to have pulmonary embolism, three were found to have aortic dissection, and 539 (84.0%) were discharged home. Within 30 days, 37 (6.6%) of those patients revisited the ED but none was related to MACE or venous thromboembolism. For patients discharged from the ED, TRO patients had a shorter length of stay (mean 14.45 vs 21.86 hours).

The vast majority of TRAD had stress testing that included MPI (N=707, 85.3%), while 71 (8.6%) underwent stress echocardiography and 51 (6.2%) underwent other risk stratification modalities, including treadmill stress testing alone, stress positron emission tomography. Seven hundred thirty-nine (89%) patients were discharged home from the ED. Within 30 days, 80 (10.5%) of those patients revisited the ED but none was related to MACE or venous thromboembolic disease.

## DISCUSSION

Patients evaluated with TRO tended to have a shorter ED length of stay, fewer hospital costs, and less exposure to radiation than traditional testing. No patient in the TRO or traditional cohort that was discharged from the hospital had a short-term adverse event, identifying that both methods are effective at safely ruling out short-term events. Given the low rate of life-threatening chest pain diagnoses and high rate of patient discharge from the ED, our study population represents a low risk group of patients.

Limited literature exists evaluating the performance characteristics of TRO as a mono-testing strategy for emergency patients. In one study by Madder et al,^28^ TRO was compared to a large cohort of ED and elective patients to evaluate its ability to detect coronary disease. TRO had similar performance characteristics to dedicated coronary CT angiography, and no patient returned for missed ACS. The control group of this study was not an ED cohort, however, and the results of this study do not directly address the evaluation of the ED patient with undifferentiated chest pain. Rogers et al^29^ prospectively evaluated TRO compared to dedicated chest CT protocol for patients in the ED presenting with acute, undifferentiated chest pain. They found no difference in total hospital length of stay, radiation exposure, or cost between groups, although their definition of length of stay included total hospital time and ED time. A lack of sample size (total N=59) likely contributed to the lower, yet non-statistically significant, rates of MACE, on-going clinical symptoms, and revisits in the TRO group at follow up. Importantly, Rogers et al did not evaluate specifically for coronary artery disease in the dedicated chest CT arm. Finally, Takakuwa and Halpern^35^ investigated the use of TRO in low-to-moderate risk ED patients with symptoms and history concerning for ACS. They used TRO to evaluate for coronary artery disease versus alternative diagnoses to explain each patient’s presentation. Ultimately 11% of their study population had a clinically important alternative diagnosis and 76% of patients with no to mild coronary disease required no further testing.

A recent meta-analysis looking at TRO compared to other diagnostic modalities for nontraumatic chest pain included 11 studies and concluded that TRO is highly accurate for coronary artery disease but associated with increased radiation exposure.^33^ In contrast to our investigation, the studies included in Ayaram et al did not exclusively enroll ED patients and did not evaluate all patients for undifferentiated chest pain with a non-invasive strategy. While their analysis adds to the literature on TRO, it did not address the clinical utility of TRO compared to other currently used diagnostic strategies for emergency patients. Despite its broad review of the available literature, it cannot be used in isolation to draw conclusions on the usefulness of TRO in the ED.

As technology changes, so does our ability to image with less radiation, less contrast volume, and less beta blockade. New imaging techniques have allowed for the improved safety profile of TRO protocols while obtaining adequate quality.^36^–^40^ In the future, prospective and randomized study of TRO vs TRAD is needed in the evaluation of undifferentiated chest pain patients in the ED. Furthermore, prospective study with actual radiation dose measurements and longer follow-up periods for MACE would be useful.

## LIMITATIONS

Limitations of this study include its short follow-up period and potential to have missed revisits, adverse events, or deaths not presenting to our own institution. A short follow-up period was chosen since the alternative to admission or ED observation for further risk stratification is short-term outpatient testing. Given the similar safety profiles between the TRO and TRAD groups, it seems our patient population was sufficiently low risk, such that further outpatient testing may have been reasonable.^41^

Because of this study’s retrospective design, we have little information regarding physician testing strategy other than clinician judgment led to testing for more for more than one etiology of chest pain in these patients. Furthermore, we have little information regarding baseline characteristics of the two groups, and as such, we cannot directly compare groups further. However, since there is no previous literature comparing TRO to a TRAD strategy in ED patients with undifferentiated chest pain, this study represents a necessary pilot investigation.

Our institution frequently uses MPI to increase diagnostic accuracy for ACS^41^ but has increasingly used CT coronary angiography when coronary artery disease is the leading diagnostic concern. Institutions that use stress testing alone or in combination with echocardiography would be expected to identify lower radiation exposure compared to their traditional testing but would still be limited by image quality and operator skillfulness.

Finally, our length-of-stay data may be biased by the fact that not all diagnostic tests were available 24 hours a day or seven days a week, and observation overnight was sometimes required to obtain further objective testing. At our institution, neither TRO nor TRAD was available after 7 p.m. and stress echocardiography was not available on weekends. While the difference in availability of specific choices in provocative testing may influence the length-of-stay advantage of TRO, less than 10% of the TRAD cohort received stress echocardiography implying that influence was minimal. While resource availability for chest pain rule-out pathways at all times would be ideal, this is not necessarily feasible in all institutions.^42^

## CONCLUSION

Undifferentiated chest pain evaluation by TRO in the ED appears to be a feasible, safe, and effective modality for excluding life-threatening causes of chest pain for low risk patients in the ED. Prospective studies evaluating the clinical utility of this diagnostic strategy are needed to further assess this approach to ED chest pain evaluation.

## Figures and Tables

**Figure:**
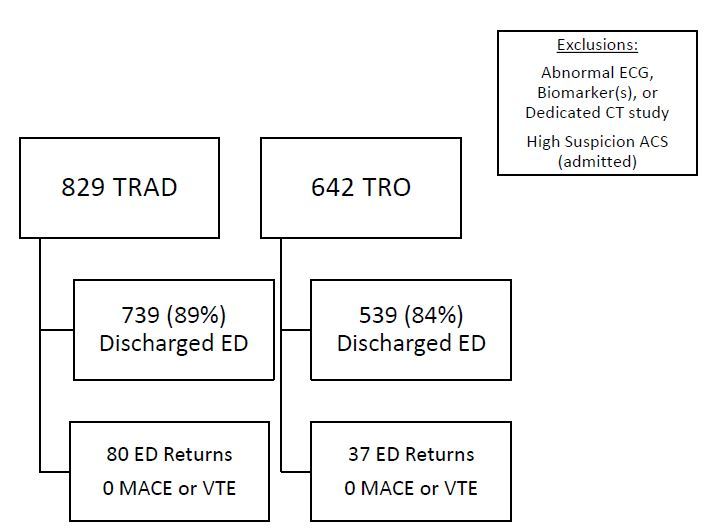
Patient summary diagram. *ECG*, electrocardiogram; *CT*, computed tomography; *ACS*, acute coronary syndrome; *TRAD*, traditional group; *TRO*, triple rule out group; *ED*, emergency department; *MACE*, major adverse cardiac event; *VTE*, venous thromboembolism

**Table 1:** Summary demographics by study group.

Demographic	TRAD N=829	TRO N=642
Age (years)	56.5 (SD 14.61)	52.3 (SD 12.04)
Gender (male)	252 (30%)	272 (42%)
Body mass index	31.8 (SD 8.04)	29.4 (SD 6.23)
Length of stay (hours) in ED	21.86 (SD 6.14)	14.45 (SD 7.54)

*TRAD,* traditional group; *TRO,* triple rule out group; *ED*, emergency department

**Table 2:** Radiation estimates (mSv) based on body mass index.

Body mass index	Myocardial perfusion imaging	CT chest
BMI<30	12.1mSv±1.2	4.5mSv
BMI 30–45	12.1mSv±1.2	8.2mSv
BMI>45	16mSv±1.6	13mSv

*CT*, computed tomography; *BMI*, body mass index
